# An Experiment and Its Result

**Published:** 1872-12

**Authors:** H. Gerhart


### ARTICLE XI.

### *An Experiment and its Result.*

**Read before the Susquehanna Dental Association, May 16th, 1872.**

BY. H. GERHART, D. D. S.

Six months ago, (Nov. 1871,) at the close of a busy day, a lad of 14 presented himself, who, twenty-four hours previous, had received upon the mouth a blow from a shinney, whereby the left lateral and central incisors of the upper jaw were dislocated, the right lateral broken off near the gum, and the right central entirely dislodged, while the lips and the gums of the upper jaw were bruised and lacerated; the parts were highly inflamed, and had an unpromising appearance.

After considering the case a few moments, remembering the recuperative power of the youth, and with an eye to preventing the contraction of the arch that would follow the loss of two teeth, and the unsightliness of a solitary central incisor, I concluded to try an experiment to replant the dislodged tooth and to pivot the broken one. The dislocated teeth he had with his own hand pressed back to their sockets.

Upon asking him for the missing central, he said one of his mates had carried it to the school-room, half a mile away. When he understood what was proposed to be done, he was off like a shot, and in a short time I had in my hand a tooth that for more than twenty-four hours had been drying in a heated school-room. This tooth appeared more unpromising than the mouth, but the lad was anxious for the experiment from the moment it was suggested.

The apex of the root was shortened and polished, the root filled with gold, and the tooth soaked five minutes in warm water, slightly impregnated with carbolic acid; the socket was carefully cleared of the coagulum and incipient granulation, and thoroughly syringed with warm water, when the tooth was slowly but firmly pressed home, and secured by a ligature to its mate; after this the parts were for ten days, several times each day, bathed with highly diluted tincture of calendulum, no other dressing being used.

The lips and gums continued inflamed and swollen for a number of days, with considerable suppuration, which was to be expected. There was not much pain, but for more than two days the patient complained that the replanted tooth felt cold, and not until near the end of the third day was it "warm and comfortable," as he expressed it.

In the process of suppuration the tooth was extruded slightly from its socket, from which two results followed—the tooth is a little more flexible and is a trifle longer than its mate, but not sufficiently so as to make it either inconvenient or unsightly.

After the lapse of two months the pulp of the broken tooth was devitalized (it had retained its vitality all this time) and the root was pivoted; and two months later a crown cavity in the replanted tooth was filled, and to this day the lad has felt no discomfort from the tooth, and to an ordinary observer there is no difference between its color and the color of its neighbors.

I have written this that we may be impressed afresh with the importance of the conservation of the natural organs, and that we may renew our trust in our most faithful ally, Indulgent Nature.—*Dental Times.*

